# Experimental Analysis of the Relationship between Textile Structure, Tensile Strength and Comfort in 3D Printed Structured Fabrics

**DOI:** 10.3390/polym15010152

**Published:** 2022-12-29

**Authors:** Jorge I. Fajardo, Marco V. Farez, César A. Paltán

**Affiliations:** New Materials and Transformation Processes Research Group GiMaT, Universidad Politécnica Salesiana, Cuenca 010102, Ecuador

**Keywords:** additive manufacturing, fabric weave, structured fabrics, wearables

## Abstract

In this article, an experimental investigation was conducted to study the effects of 3D printed structured fabrics on the tensile strength of two additive manufacturing technologies: (i) fused deposition modeling (FDM); and (ii) stereolithography (SLA). Three types of structured fabrics were designed in a linked fabric structure, which resembled the main characteristics of a conventional textile. Through computer-aided design (CAD), the textile structures were sketched, which, in a STL format, were transferred to 3D printing software, and consequently, they were printed. The specimens were subjected to tensile tests to analyse the behaviour of the linked structures under tensile loads. The results obtained indicated that the elements structured in a linked fabric pattern showed a statistically significant effect between the design of the 3D printed structured fabric and its tensile strength. Some important properties in textiles, fabric areal density, fineness (tex) and fabric flexibility were also analysed. This study opens an important field of research on the mechanical resistance of textile structures manufactured by 3D printing, oriented for applications in wearables that have a promising future in the fields of medicine, aerospace, sports, fashion, etc.

## 1. Introduction

3D printing, an additive manufacturing technology, has increased research interest in recent years, because it reduces raw material waste and offers shorter production time, ease of creating complex designs, the possibility of using biodegradable materials and excellent cost−benefit ratios [1,2]. Thanks to these advantages, it is applied in various fields of science and technology. Applications are reported in industries including aerospace, medicine, design, manufacturing, and fashion [3,4,5].

The textile industry records sales of over 450 billion dollars worldwide, making it one of the largest, but at the same time one of the most polluting, due to its production volume, its industrial processes and the durability of worn textiles, which are disposed of in dumps or incinerated [3,6]. 3D printing contributes to sustainability with the use of biodegradable materials such as polylactic acid (PLA), which can also be recycled, reducing environmental pollution and increasing the durability of products [7,8]. With 3D printing, even a designer will no longer need to manufacture the textile to sell it but can sell the digital design, which can be customizable and scalable, providing the ease of being able to print it at home according to its use, or distribute it. It can also have a major impact on supply chains, reducing manufacturing steps and improving distribution and decentralized production [8]. 3D printing has converged with other areas of science, favouring the creation of so-called smart fabrics (wearables). Wearables can detect the heart rate, body temperature, skin tone, etc., which can be altered by environmental phenomena or related to psychological situations. These garments transmit data to an intelligent system that emits protection activation signals to the user [9].

Fused deposition modeling (FDM) is the most widely used technology in the field of additive manufacturing in general, mainly due to the economic costs of both printing equipment and thermoplastic filaments, and it is also the simplest additive manufacturing technology, making it quite accessible to the general public [10]. However, the main disadvantage of this technology is its low-quality finish. It also requires support structures, so that 3D printed objects can have certain characteristics such as cantilevered elements. These supports often need to be broken mechanically or can also be dissolved in certain detergents [8,10]. Another disadvantage also results from printing lines between layers being visible, and these are very susceptible to delamination, which can also be caused by temperature fluctuation inside the printer during printing [8]. On the other hand, there is stereolithography (SLA), which is one of the fastest 3D printing technologies and with the best surface finish quality for a 3D printed product. However, one of its major disadvantages is its cost, in terms of both printing equipment and printing materials, which are very expensive and limited. In addition, similar to FDM technology, it requires support elements that secure the 3D object for its construction and are removed after the process is completed, which can reduce the quality of the finish in some cases [8,11].

In this boom in research, the literature reports studies that evaluate the effects of the properties of textiles on tensile stresses and deformations of components made with polylactic acid (PLA) printed on polyethylene terephthalate (PET) fabrics [12]. Other studies evaluate the effect of the architecture of fabric weaves on the elastic behaviour of fabrics [13]. Studies on impact resistance and electrical conductivity in structured tissues obtained by selective laser sintering (SLS) are also reported, with the possibility of controlling their structural rigidity [14]. Other studies report analysis of compressive stresses in linked and non-linked elements in confinement packing, concluding that the linked elements offer greater resistance to breakage and this type of structure presented a 10% breakage of the links [15]. In addition, studies related to topologically interlocked elements are reported, in which it has been proven that geometric modifications provide a way to manipulate deformation mechanisms, increasing the performance of materials and increasing load and impact capacity [16,17]. For the literature about 3D printing of textile-like structures, studies have been carried out to evaluate different novel patterns and construction methods for the area of clothing and related to this. In this area, they proposed to print structural units and then assemble them to form functional textiles, also allowing creating designs with new functionalities that exceed those of traditional textiles [18,19,20]. On the other hand, in studies about 3D printing in combination with textile fabrics, there are researches which report studies about the influence of a polymer coating on different textile fabrics on the adhesion of hard and soft PLA-based printed three-dimensional elements. They found that polymer coating increases the adhesion for hard PLA while soft PLA on some textiles adheres better without the need of coating [21]. In the same line of investigation, studies have also been conducted evaluating the adhesion of printed TPU elastic patterns on conventional textile fabrics, finding that these patterns significantly influence the mechanical strength [22,23]. A similar investigation examined the possibilities of printing 3D shapes directly on textile fabrics, using soft PLA which was printed on fabrics of cotton, wool, viscose and a polyester web, observing that the adhesion of PLA printing on wool increases slightly due to the rough surface of the fabric. However, only for the polyester web, the printed material is actually attached to the fabric, as the molten PLA flows around individual threads and encloses them [24]. Other studies analyse polyurethane composites printed on polyester fabrics, where it was found that the patterns that were printed significantly influenced the mechanical strength properties analysed [25]. Although 3D printing is still far from being able to provide the main characteristics of a 3D printed textile product, this technology proposes to integrate a modular system that contributes to the ease of design and customization while promoting environmental sustainability, since the textile industry is one of a few industries today that have not yet developed materials and production systems that reduce CO_2_ emissions or recycling of worn textiles [7,18]. Some designers have already proposed novel 3D printed designs such as Israeli designer Danit Peleg [2], which demonstrate the immense potential that this technology is being introduced to the textile industry.

In recent years, man-made auxetic materials with desired performance have been designed, which have potential applications in the fields of textile wefts, biomedical engineering, sensors, and new functional structures. The possibility of manufacturing this type of structures through 3D printing is an emerging research topic, because they present negative Poisson’s ratio behaviour which provides materials with better energy absorbing and shock resistance capabilities as compared to conventional materials. Investigations on the mechanical properties of 3D printed chiral auxetic geometries of various sizes are reported. Small-scale samples were 3D printed and tested under compression and tension to ascertain their strength and deformation characteristics. The re-entrant chiral cell size has been shown to affect the mechanical properties of the re-entrant chiral auxetics [26]. Similarly, several geometries of re-entrant chiral auxetic (RCA) structures created using 3D printing have been evaluated. Each geometry has been tested under tension, compression and bending to investigate the effect of varying cell size on the stress−strain behaviour, Poisson’s ratio and energy absorption properties [27]. On the other hand, the hybrid structure takes more load than the auxetic structure, which has been experimentally demonstrated. The ultimate tensile strength (UTS) of the hybrid structure becomes 32% more than that of the auxetic structure. These results have been validated using the finite element results [24]. Experimental campaigns to obtain stress–strain curves and local strains in the two principal directions by the standard experiments are reported. The stress–strain curves and local strains have been used to obtain the mechanical properties, including Young’s modulus, yield strength and Poisson’s ratio of the auxetic structure [28].

As can be seen, additive manufacturing technologies applied to the clothing industry have made significant advances in recent years, and the products obtained are intended to replace conventional textiles [7,29]. Although there are studies that deepen in the analysis of the mechanical behaviour of honeycomb, grid or auxetic textile structures manufactured by 3D printing or patterns of printed shapes on textile fabrics, these studies have not analysed the mechanical properties and textile comfort in 3D printed structured fabrics in a linked fabric pattern, denoting that there is scarce information on the study of this type of 3D structures. The present study aims to experimentally evaluate the effects of the designs of three textile structures obtained by different additive technologies, evaluating the tensile and comfort properties and comparing their mechanical resistance with auxetic and conventional textile structures.

## 2. Materials and Methods

### 2.1. Materiales

For fused deposition printing (FDM), a high-molecular-weight polylactic acid (PLA) filament was used, with a tensile strength of 49 MPa, a Young’s modulus of 2.8 GPa, a Poisson’s ratio of 0.4, a density of 1.24 $g/{\mathrm{cm}}^{3}$, a melt flow rate of 7–9 g/10 min and a diameter of 1.75 mm, provided by SUNLU (Irvine, CA, USA). On the other hand, for stereolithography (SLA) printing, standar UV liquid resin with a photo initiator was used, with a density of 1184 $g/{\mathrm{cm}}^{3}$, a tensile strength of 23.4 MPa and a shrinkage of 3.74–4.24% provided by ANYCUBIC LCD SLA (Shenzhen, China).

### 2.2. Manufacturing Process

**Figure 1:**
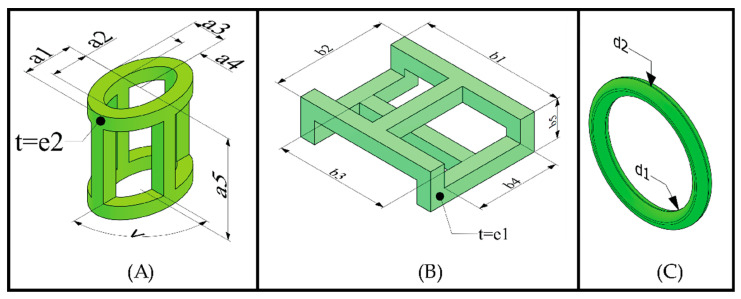
Designs of the elements that are composed of the different textile structures. (**A**) Cylindric element; (**B**) prismatic element; (**C**) annular element.

**Figure 2:**
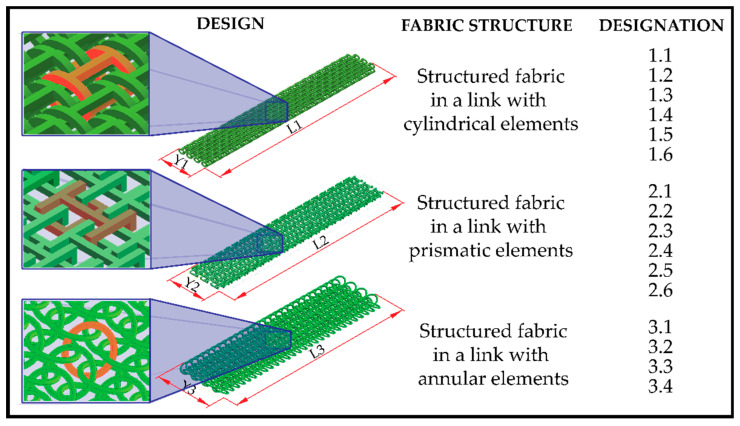
Design and designation of the specimens structured in a linked fabric structure for the research study.

Structured fabrics were designed in a linked fabric pattern, with a similarity to traditional textiles. CAD designs were transferred to 3D printing software (Ultimaker cura for FDM and Grabcad for SLA) in the STL format. A total of 16 specimens were fabricated and divided into three groups: (i) 6 specimens for cylindrical element structures in woven link; (ii) 6 specimens for prismatic element structures in woven link; and (iii) 4 specimens for annular element structures in a woven link. The dimensions of the unit elements and the dimensions of the specimens are shown in Figure 1 and Figure 2 and Table 1. The cylindrical element specimens consisted of 161 elements, arranged in repeats of 3 and 4 elements per transverse row. The prismatic element specimens consisted of 143 elements, also arranged in repetitions of 3 and 4 elements per transverse row. In the specimens with prismatic elements, it should be noted that the longitudinal rows at the edge were composed of prismatic elements with dimension (b2 = 4 mm), as explained graphically in Figure 1B. These edge elements were arranged in this length division to avoid the entanglement of the prismatic elements and to establish a width of 31 mm in the specimen. The annular element specimens were composed of 224 elements arranged in transverse rows of 4 elements. The structures of cylindrical and prismatic elements were printed by FDM, with a speed of 80 mm/s, a layer thickness of 0.15 mm, a temperature of 215 °C and 100% infill on an Original Prusa i3 MK3S+ direct extrusion printer with a 0.4 mm nozzle diameter. While the annular elements were printed by SLA, with a layer height of 0.005 mm, an exposure time of 12 s and a raising speed of 65 mm/min on a Stratasys printer with a precision of 1 mm, object studio^TM^ intuitive software 3D and 16 micron print quality.

### 2.3. Tensile Tests

Tensile tests were performed on an AGS-X 300kN universal materials testing machine (SHIMADZU, Tokyo, Japan). The machine was equipped with a 20 kN load cell and screw type flat grips (machine attachment), with the flat grip face dimensions of 35mm in width and 25 mm in length, were used to hold the specimen. A TRViewX digital video extensometer (SHIMADZU, Tokyo, Japan ) with a camera having an FOV of 120 mm (f25 mm) was used to record the fracture sequence of the linked fabric structure in a video. The accuracy of the video extensometer was in accordance with ISO 9513 class 0.5. Quasi-static tensile tests were performed for isotropic materials in the machine direction. Tensile properties were obtained according to ASTM D638. According to the recommendations of the standard for type III specimens, the dimensions detailed in Table 1 were used. The test speed was 10 mm/min, which is within the recommendations of the standard. The distance between grips was 120 mm for cylindrical and prismatic specimens, while for annular specimens, the distance between grips was 65 mm. The test was carried out at an air-conditioned ambient temperature of 20 °C with consolidated humidity. The tensile results were obtained using TRAPEZIUMX software (SHIMADZU, Tokyo, Japan) with a data-recording rate of 5 kHz, a sampling rate of 300 kHz and a single test mode. The results obtained from the tests were the deformations, maximum stresses and Young’s moduli.

### 2.4. CAE Modeling

**Figure 3:**
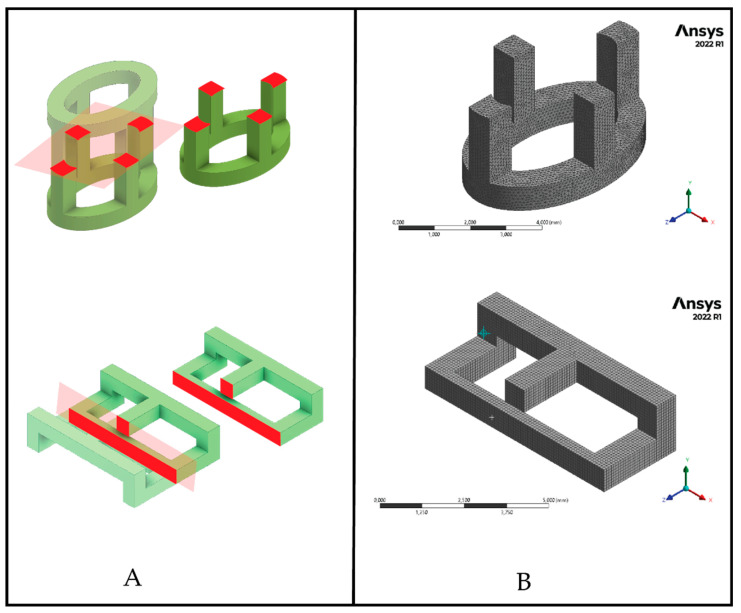
Geometric simplification of the cylindrical and prismatic elements (**A**) and meshing of the simplified models (**B**).

The CAE modeling was carried out in software ANSYS 2022 R1. The process included pre-processing in which the symmetry of the load and the support geometry was used to simplify the model, as can seen in Figure 3A. The material properties were input in the CAE software according to the properties described in Section 2.1. A mesh convergence analysis was performed with an average mesh element quality of 0.845 and an average Skewness of 0.218, obtaining a mesh with 178,273 nodes and 122,166 tetrahedral elements, and the average element size was 0.1 mm for the cylindrical links, while for the prismatic links an average mesh element quality of 0.998 and an average Skewness of 0.012 were obtained with 60,534 nodes and 12,496 tetrahedral elements, having an average element size of 0.1 mm. The boundary conditions were set as shown in Figure 3A. In post-processing, a von Mises equivalent stress analysis was performed to identify areas of stress concentration.

### 2.5. Textile Properties

Flexibility analyses were carried out, measuring the specimens in the contracted and extended states according to ASTM D3773. The measurements were performed with a Mitutoyo digital caliper (CONIC TOOLS, Fujian, China), with a precision of 0.1 mm. The ASTM D3776 standard was used to measure the fabric areal density, using the densities of PLA and resin indicated in Section 2.1. The yarn fineness (yarn count) was calculated based on the direct system “TEX” and the indirect system (new metric count). The linear fabric density and yield were obtained from the fabric areal density.

## 3. Results

### 3.1. Tensile Tests Results

**Figure 4:**
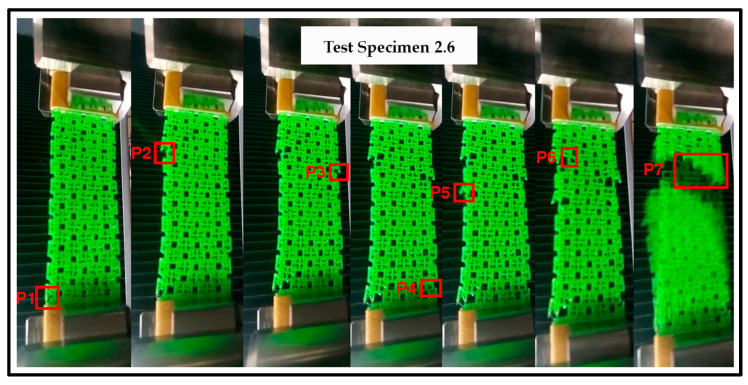
Random breakage sequence of the specimen 2.6.

**Figure 5:**
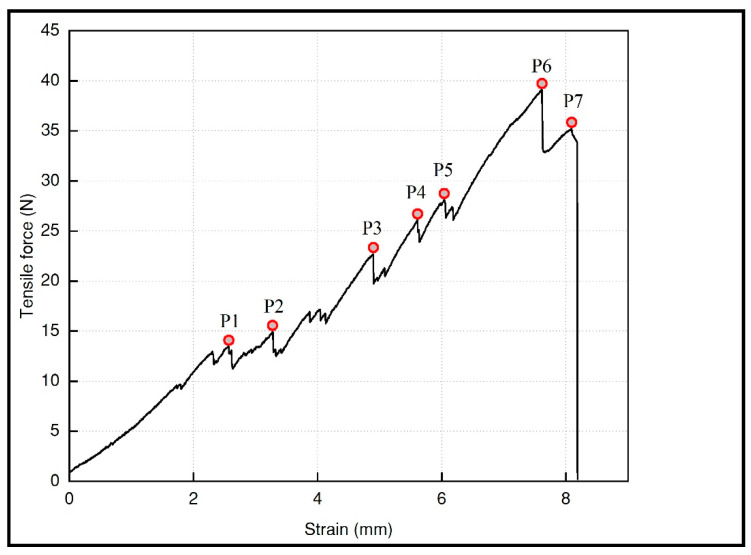
Fluctuations in the loads recorded in the force−deformation curve of the specimen 2.6.

Due to its structure in a linked fabric patern, the breakage of the test pieces showed load fluctuations that were recorded in the force−deformation curves, as the random breakage of the links in the test piece occurred, as shown in Figure 4 and Figure 5. This type of stochastic fracture is attributed to stick-slip mechanisms typical of dry friction [30]; however, the information about studies that evaluate the effects of 3D printed strucutural fabrics on the tensile strength is scarce, and no information has been found to compare this behaviour with similar structures subjected to traction. For this reason, in this study a video extensometer was used to record random sequences of fracture of the links, and the stresses in each peak were calculated, correlating it with the values of the force−strain curve.

**Figure 6:**
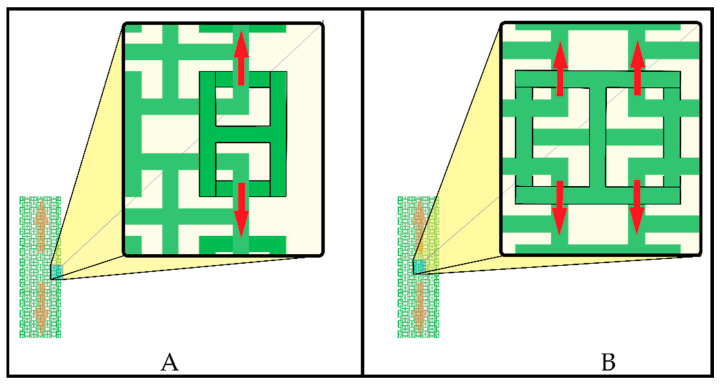
Stress conditions: (**A**) edge elements; and (**B**) inner elements.

In Figure 4, we can see that the crack initiation occurred in the elements that were on the lateral edges (P1, P2, P3, P4, and P5) and then the crack front propagated through the adjacent links through the material until total failure (P6 and P7), so it is suggested that there is no homogeneous stress distribution for each element that makes up the specimen, since the elements, being linked, had certain degrees of freedom that were redistributed as they deform. Figure 6 schematically describes the elements of the edge of the specimen and stress conditions that are different from the elements that are inside. The edge elements in Figure 6A are under single stress, while the inner elements in Figure 6B are under double stress. Therefore, the extreme elements are subjected to higher stresses when they are in tension, and they tend to fail prematurely as shown in Figure 4.

**Figure 7:**
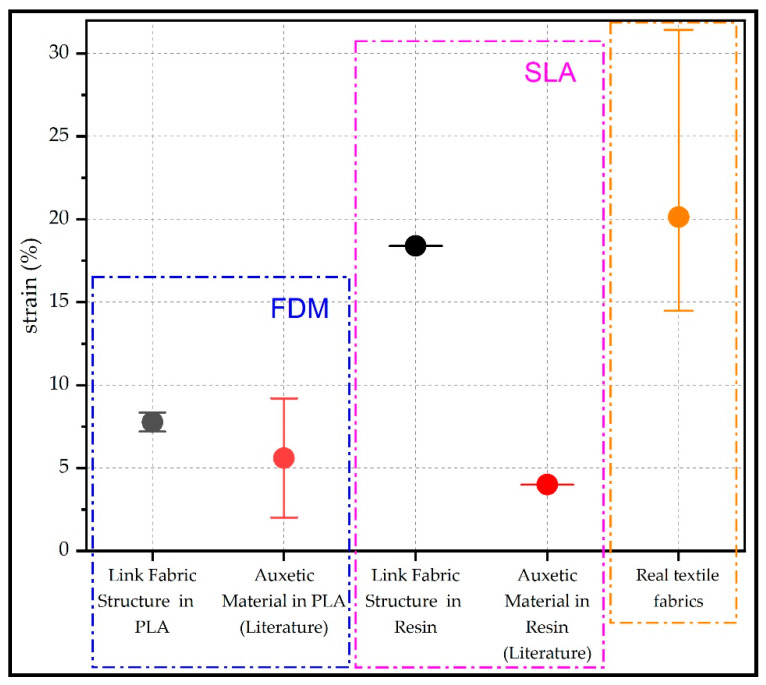
Comparison between the strains of the link fabric structure, the auxetic material and the real textile fabrics.

Local stresses were determined and then averaged to obtain a resultant stress. Table 2 shows the average stress, strain and Young’s modulus for the cylindrical element specimens (1.1–1.2), circular element specimens (2.1–2.6) and annular element specimens (3.1–3.4), revealing that the structured fabric formed by prismatic elements had higher tensile stresses compared to the structured fabric formed by cylindrical and annular elements. According to the literature consulted, studies have been found that evaluate mechanical properties in 3D printed auxetic materials, which have geometrical and structural characteristics belonging to the group of mesh structures, i.e., structures composed of elements joined without linkage. The literature reports the maximum tensile stress of 1.5 MPa using PLA printed chiral auxetic geometries of various sizes, obtaining strains in the range from 1.5% to 7.5%; however, this higher strain of 7.5% is obtained by reducing the stress, since the maximum stress of 0.58 MPa was obtained for this type of structure [26]. Another study in auxetic materials reports tensile stresses of 1.6 to 2.7 MPa with strains in the range from 0.5% to 9.2% [27]. Furthermore, the regular honeycomb hybrid auxetic structure has the maximum stresses of 22.6 MPa with strains in the range from 1% to 2% [24]. For cellular auxetic structures printed on SLA based on honeycomb structures, stresses of 6 MPa and 9 MPa with strains in the range from 2% to 4% are reported [28,31].The results of this study are within the range of stresses reported in the literature. However, structures fabrics showed major strains (8.34% for PLA-printed cylindrical element structures and 18.38% for SLA-printed annular element structures). Considering that the structures studied in the literature are auxetic materials (mesh materials), since the structures studied in this paper are woven link fabrics, it can be analysed that the woven link fabrics offer such strains at similar tensile stresses compared to auxetic materials printed in FDM. For SLA, we can see that the strain is higher compared to for auxetic materials printed in that technology, as shown in Figure 7.

In addition, a literature search was conducted on the mechanical strength of conventional textiles, which are mentioned below. They analysed the mechanical properties in the weft and warp directions of cotton and elastane−cotton textile fabrics with a tex of 29.5 each. They obtained the maximum tensile strengths of 608 N in the warp direction and 170 N in the weft direction, with extensions of 14% and 32%, respectively [32]. Another study evaluated the mechanical properties of auxetic textile fabrics woven in various patterns using low-stiffness monofilament polyurethane and polyester cords and high-molecular-weight polyethylene multifilament and PET yarns. The maximum tensile strengths of up to 5000 N in the weft direction and 3000 N in the warp direction were obtained with 20% elongations [33]. In addition, other investigations reported mechanical properties of cotton fabrics with tex 30 and cotton/bamboo fabrics with tex 30 were evaluated, obtaining the maximum tensions of 559.09 N and 581 N for cotton and cotton/bamboo fabrics, respectively. The maximum elongations were 22.95% in the warp direction and 16.92% in the weft direction [34]. As can be seen, there is a clear difference in that conventional textiles have better mechanical resistance properties in comparation to those studied here, as can be seen in Figure 7.

To determine if there were statistically significant differences between the designs of the proposed 3D printed structural fabrics and the tensile strength, a one-way ANOVA was carried out, which allowed us to compare the stresses of the three groups of 3D printed textile structures to check if the stress results of one test and another were significant or not. The statistical analysis was performed using STATGRAPHICS 19 software (Statgraphics Technologies, Inc., The Plains, VA, USA). The assumptions of normality of the data and homoscedasticity were verified. Two hypotheses were proposed: (i) for the null hypothesis, there is no significant difference between the mean stresses of one textile structure and another; (ii) for the alternative hypothesis, there is a statistically significant difference between the mean stresses of one textile structure and another. The first column in the ANOVA table (Table 3) shows the source factors of variation corresponding in our case study to the stress results between groups of textile structures and within groups. In the second column of Table 3, the sums of squares of each specimen stress test together with the total of all specimen tests within the sources are reported. In the third column of Table 3, the degrees of freedom based on the stress tests for between groups and within each group are reported. In the fourth column of Table 3, we reported the mean square which is the sum of the squares divided by the corresponding associated degrees of freedom. In the penultimate column of Table 3, we reported the F-ratio, which is the mean of the square between groups divided by the mean of the source squares within each group and in the last column we show the *p*-value, which evaluates the validity of the null hypothesis with a 95% confidence interval.

The ANOVA analysis was performed, which broke down the variance of the tensile stress into two components: a between-group component and an intra-group component. Since the *p*-value of the F-test is less than 0.05, there was a statistically significant difference in the mean tensile stress between the different 3D printed structural fabrics, at a 95.0% confidence interval. The summary of the analysis is reported in Table 3. This means that the geometric configuration of the elements that make up each of the structured fabrics influences the tensile strength of the overall structure.

**Figure 8:**
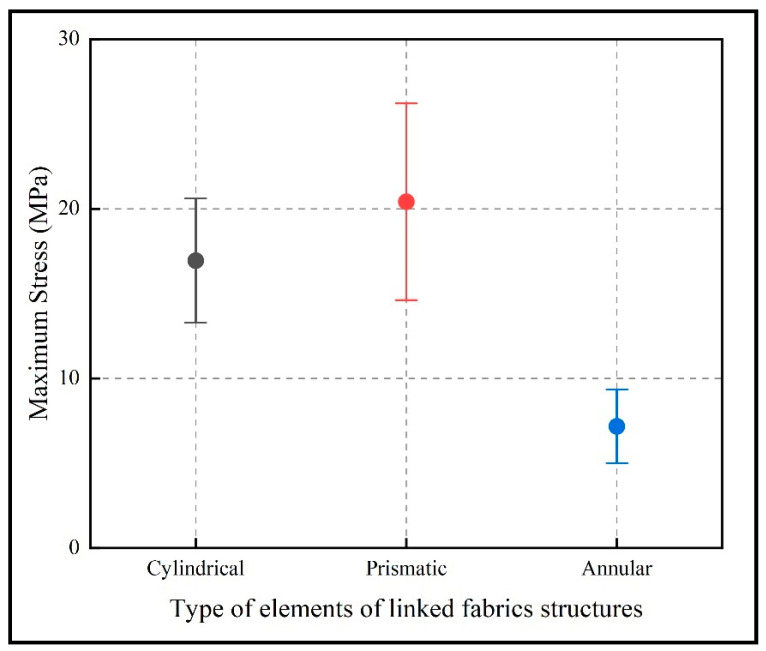
Average stresses of the different structures.

Figure 8 shows that the prismatic element structures had the highest tensile stress compared to the cylindrical elements, since both structures were composed of the same manufacturing material, while the annular element structures had the lowest tensile stress due to the properties of the photosensitive resin. This stress difference between the cylindrical and prismatic elements is due to the geometrical configuration of the elements, which in the case of prismatic elements presented higher tensile strength, as shown in the CAE modeling in Figure 8.

**Figure 9:**
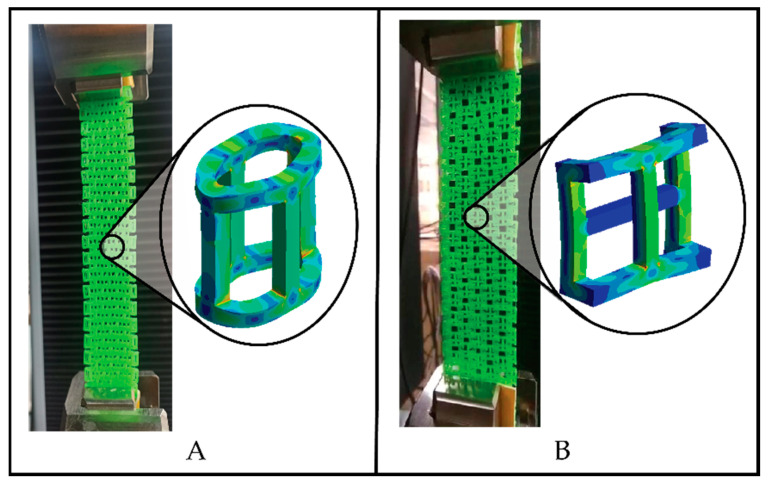
Analysis by CAE modeling of the stresses in the different elements that constituted the 3D printed textile structures. (**A**) cylindrical elements; and (**B**) prismatic elements.

**Figure 10:**
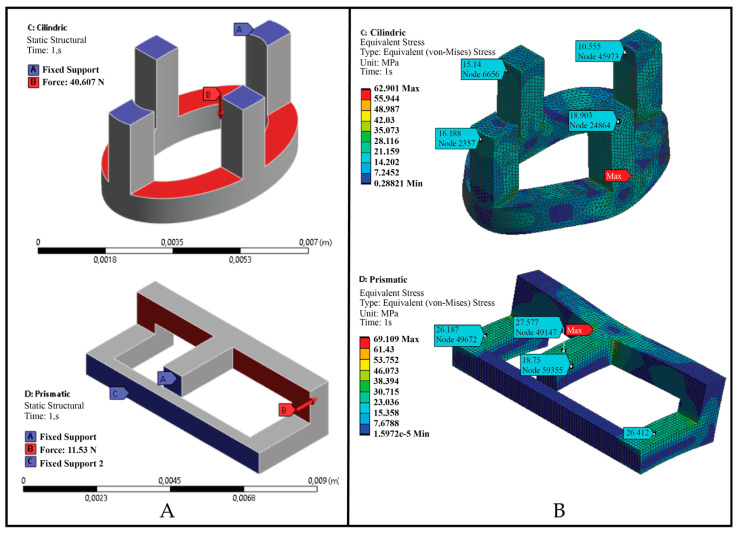
Modeling using CAE software for cylindrical and prismatic elements. (**A**) pre-processing; and (**B**) equivalent von Mises stress.

Figure 9A,B shows schematically the stress concentrators when subjected to tensile loads, while Figure 10 shows a modeling using CAE software, which allowed identifying the stress distribution in each individual element of the strongest structures. The simulation was carried out on a single element in order to be able to appreciate the stress concentration in the individual elements, since the failure of the specimen depended on the rupture of these unitary elements, as shown in Figure 4 and Figure 5.

Figure 10 presents the meshing and the report of the equivalent von Mises stress. As observed in the CAE modeling results, there was a great similarity in the stress distribution with the experimental results. They also agreed that links with a prismatic geometry had a higher tensile strength than cylindrical elements.

### 3.2. Fracture Zone Morphology

**Figure 11:**
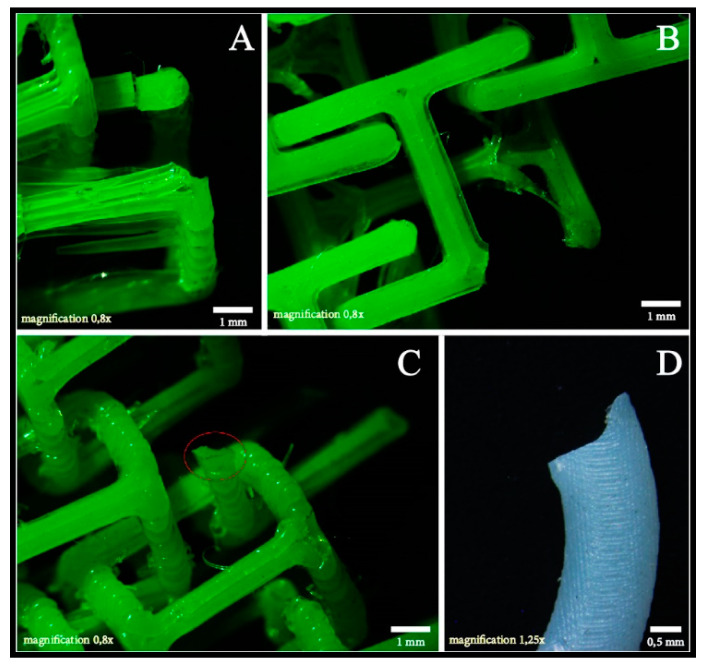
Microscopic analysis of the fracture zone. (**A**) stress concentration zone for cylindrical elements; (**B**) stress concentration zone for prismatic elements; (**C**) delamination for cylindrical elements; and (**D**) brittle fracture for annular elements.

A microscopic analysis of the fracture zones of the specimens was performed. Figure 11A,B shows that the link breakage occurred in the stress concentration zone, reaffirming the results of the CAE analysis (Figure 10). In Figure 11C, there was a complete separation of the printed layers as a result of a delamination, which consequently produced a breakage of that point in the element and evolved into the total breakage of the specimen. Finally, we can see in Figure 11D that the failure of the annular elements occurred from a brittle fracture, due to the rigidity of the resin used in the SLA additive technology.

### 3.3. Textile Characteristics of 3D Printed Fabrics

**Figure 12:**
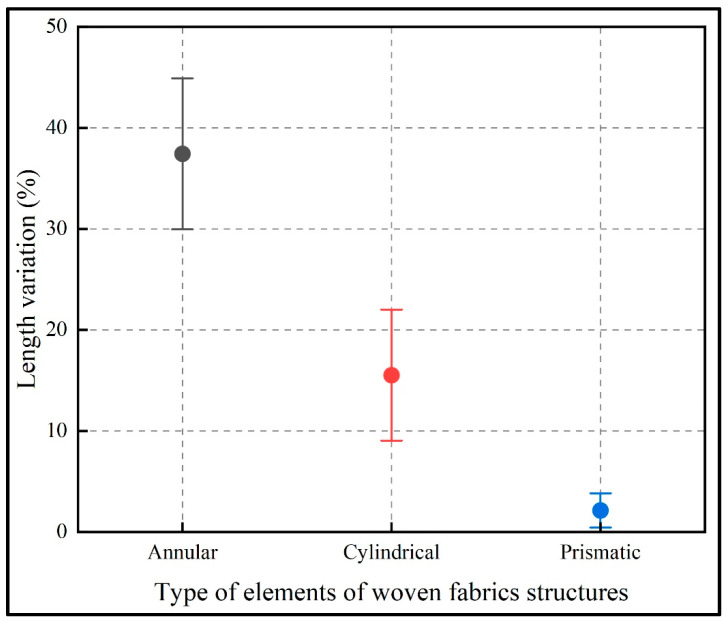
Average variation of 3D printed structured fabrics length.

The main textile characteristics were analysed: flexibility (extension and contraction capacity), fineness, fabric areal density and texture. The results are presented in Figure 12, where the percentage of variation in length of each link design (annular, prismatic and cylindrical) is detailed. The flexibility analysis is based on the fact that the textile must withstand repeated stretching without decreasing its breaking strength and the degree of flexibility determines the ease with which the textile can be bent and stretched. Being a structure that is not composed of yarns such as a traditional textile, but of linked elements provides a certain degree of flexibility, mainly due to the space that exists between one element and another, which, multiplied by the number of elements in the structure, provides the capacity for mobility in the direction of stretching, thus producing an extension of the entire structure and shrinkage in the opposite direction. Based on the above discussions, measurements were made in the two states of the specimen (stretched and contracted), thus obtaining the percentage variation of the length of the specimen as shown in Figure 12. It was observed that the structure of linked fabrics composed of annular elements had the best extension variation with a percentage of 37.43%, which implied greater flexibility and increased the capacity for making and adapting to the morphology of the human body, since the elements links that make up this type of structural fabric allow greater mobility, enabling it to adapt to different shapes and emulating a traditional textile in a better way.

The fineness was carried out in the direct “tex” system and in the indirect system (Table 3). Analogous to the textile numbering, the analysis of the weight property and its textile yield was carried out, as shown in Table 4. Textile yield is the measure of the fabric length per unit weight [35]. The weights for all textile structures were high, which means that these printed textile structures were located in the range of heavy textiles/fabrics [36].

**Figure 13:**
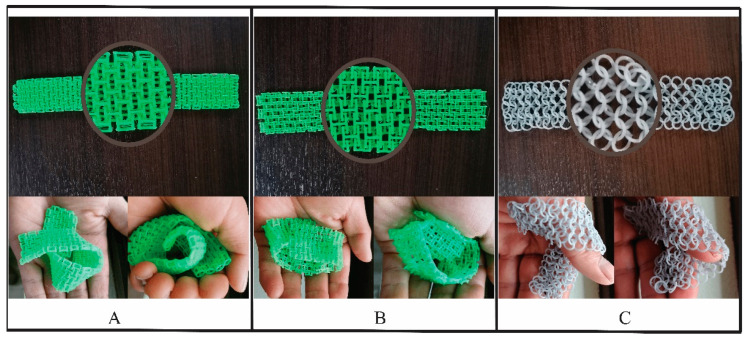
Visual, tactile and comfort inspection analysis. (**A**) cylindrical elements; (**B**) prismatic elements; and (**C**) annular elements.

In Figure 13, the analysis of the texture for the different printed textile plots is presented, through a visual, tactile and comfort inspection. In Figure 13A, the 3D printed structural fabric composed of cylindrical elements showed a homogeneous tactile texture, due to the structuring of cylindrical elements, which can be infinitely extended. For the tactile and comfort, the structural fabric was a little shiny on the print bed side and softer on the opposite side, and the structure was very malleable, very flexible and very similar to a traditional textile. In Figure 13B, the grid of prismatic elements is seen, which had a homogeneous geometric visual texture, due to the structuring of prismatic elements, which can be extended infinitely. For the tactile comfort, it felt rough, and the structure had great flexibility and was very malleable, like a traditional textile. Figure 13C shows the plot of annular elements, which had a homogeneous geometric visual texture, since the linkage between the rings maintained an aspect of repetition that can be extended infinitely. For the tactile comfort, the sensation was very soft and smooth. The structure had great flexibility and was very malleable and very similar to a traditional textile of great comfort.

## 4. Discussion

The trends of 3D printed fabrics are divided into two large groups: one group with a linked structure (with a link) and one group with a mesh structure (without a link) [2]. However, mesh structure textiles are the most studied in the literature. These textile structures differ from traditional textile structures that are woven (openwork, mesh, and non-woven), because they are manufactured by weaving threads and are made of either natural, synthetic or artificial yarns/filaments.

3D printing has printing parameters that directly and significantly influence the quality of the product to be manufactured, so it is especially important to know and control these parameters, considering the printing material and technology. A 3D printed element, when subjected to an external load, can fail in the areas parallel to the printed layers, producing delamination which is the effect of separation of layers deposited on top of the other. FDM technology in the textile industry is ideal, due to the ease of printing and lower costs compared to other additive manufacturing processes in the textile field. Our results obtained in Table 1 are very comparable and are within the range of results of other research studies that have made prints of PLA structures on textile fabrics [12]. However, as has been analysed, our results can be improved, designing geometric configurations that reduce stress concentrations and improve mechanical resistance.

The filament fineness as well as the fabric areal density are important textile parameter for textile manufacturing, since by means of this the textile performance for the realization of a garment is obtained.

## 5. Conclusions

The linked structures that made up the 3D printed fabrics analysed in this study had statistically significant effects on the tensile strength, due to the geometric conditions that altered the mechanical properties of the elements in a linked condition. In terms of mechanical strength properties, the 3D printed structures in a linked fabric structure with prismatic elements showed better tensile results compared to the other two structures analysed. According to the analysis, the strength of the other structures can be improved by means of a geometric configuration that reduces the stress concentrations in the linked elements. Printing using SLA technology supplies better textile characteristics such as softness and texture, providing comfort to the structure. However, its tensile strength was lower than FDM printed cylindrical and prismatic patterns. It is necessary to further conduct the study of geometries that minimize stress concentrations and that offer a compromise of resistance and textile comfort. With the present study, it is envisaged that 3D printed fabrics can come to resemble traditional clothing, in terms of resistance, texture, flexibility and comfort, due to the wide range of materials with which it can be experimented and also to the use of advanced CAD design software, which provides tools for complex designs.

## Figures and Tables

**Table 1:** Dimensions of unit elements and the specimens.

Design	Dimension (mm)							Specimen	Dimension (mm)	
Cylindrical element	a1	a2	a3	a4	a5	e1	<	1.1–1.6	L1	Y1
	3	2.2	2	1.2	6	0.8	90°		163	27
Prismatic element	b1	b2	b3	b4	b5	e2		2.1–2.6	L2	Y2
	7.5	6	5.9	4.4	2	0.8			162	31.4
Annular element	d1	d2						3.1–3.6	L3	Y3
	7	5.4							100	30.5

**Table 2:** Average tensile properties of tested fabrics.

Designation	Maximum Stress	Strain	Young’s Modulus
	(MPa)	(%)	(MPa)
1.1–1.6	16.94 $\left(\pm 3.17\right)$	8.34 $\left(\pm 3.46\right)$	217.84 $\left(\pm 66.08\right)$
2.1–2.6	20.41 $\left(\pm 5.02\right)$	7.19 $\left(\pm 1.61\right)$	265.25 $\left(\pm 19.89\right)$
3.1–3.4	6.88 $\left(\pm 1.23\right)$	18.38 $\left(\pm 6.95\right)$	36.89 $\left(\pm 5.92\right)$

**Table 3:** ANOVA results for tensile stress.

Source	Sum of Squares	Df	Mean Square	F-Ratio	*p*-Value
Between-group	357.362	2	178.681	12.83	0.0008
Intra-group	181.099	13	13.9307		
Total (Corr.)	538.461	15			

**Table 4:** Fineness and fabric areal densities of 3D printed fabric specimens.

Specimen	Direct System “tex”	Indirect System	Fabric Areal Density	Yield
	$\left(N^{m}\right)$	$\left(N_{i}\right)$	$\left(g/m^{2}\right)$	$\left(m/g\right)$
Structured fabrics in a link with cylindrical elements	$4987\left(\pm 52\right)$	0.20	$1533.11\left(\pm 15.98\right)$	28.60
Structured fabrics in a link with prismatic elements	$4030\left(\pm 78\right)$	0.24	$784.06\left(\pm 15.17\right)$	40.61
Structured fabrics in a link with annular elements	$3190\left(\pm 23\right)$	0.31	$811.52\left(\pm 5.85\right)$	40.46

## Data Availability

Data is contained within the article.
